# Dynamic Parameterization and Optimized Flight Paths for Enhanced Aeromagnetic Compensation in Large Unmanned Aerial Vehicles

**DOI:** 10.3390/s25092954

**Published:** 2025-05-07

**Authors:** Zhentao Yu, Liwei Ye, Can Ding, Cheng Chi, Cong Liu, Pu Cheng

**Affiliations:** 1 Navy Submarine Academy, Qingdao 266000, China; cheng.chihhu@163.com (C.C.); gaoxiaocong1121@163.com (C.L.); chengpu@nudt.edu.cn (P.C.); 2 Qingdao Innovation and Development Center, Harbin Engineering University, Qingdao 266500, China; ylw322527007@hrbeu.edu.cn; 3 PLA Unit 92677 of China, Qingdao 266500, China; dican011@126.com

**Keywords:** aeromagnetic compensation, unmanned aerial vehicles (UAVs), calibration flight, Tolles-Lawson equation

## Abstract

Aeromagnetic detection is a geophysical exploration technology that utilizes aircraft-mounted magnetometers to map variations in the Earth’s magnetic field. As a critical methodology for subsurface investigations, it has been extensively applied in geological mapping, mineral resource prospecting, hydrocarbon exploration, and engineering geological assessments. However, the metallic composition of aircraft platforms inherently generates magnetic interference, which significantly distorts the measurements acquired by onboard magnetometers. Aeromagnetic compensation aims to mitigate these platform-induced magnetic disturbances, thereby enhancing the accuracy of magnetic anomaly detection. Building upon the conventional Tolles-Lawson (T-L) model, this study introduces an enhanced compensation framework that addresses two key limitations: (1) minor deformations that occur due to the non-rigidity of the aircraft fuselage, resulting in additional interfering magnetic fields, and (2) coupled interference between geomagnetic field variations and aircraft maneuvers. The proposed model expands the original 18 compensation coefficients to 57 through dynamic parameterization, achieving a 22.41% improvement in compensation efficacy compared with the traditional T-L model. Furthermore, recognizing the operational challenges of large unmanned aerial vehicles (UAVs) in conventional calibration flights, this work redesigns the flight protocol by eliminating high-risk yaw maneuvers and optimizing the flight path geometry. Experimental validations conducted in the South China Sea demonstrate exceptional performance, with the interference magnetic field reduced to 0.0385 nT (standard deviation) during level flight, achieving an improvement ratio (IR) of 4.1688. The refined methodology not only enhances compensation precision but also substantially improves operational safety for large UAVs, offering a robust solution for modern aeromagnetic surveys.

## 1. Introduction

Aeromagnetic detection is a geophysical technique that measures variations in the Earth’s magnetic field using magnetometers mounted on aircraft. The primary objective of aeromagnetic surveys is to investigate subsurface geological structures and identify mineral resources [1,2,3]. This is achieved by equipping aircraft with magnetometers and associated instrumentation, which collect geomagnetic data along predefined flight lines at specified altitudes over the target area. However, the aircraft itself, being composed of metallic materials, possesses inherent magnetic properties. As it maneuvers through the geomagnetic field, it generates an interference magnetic field that can significantly degrade the accuracy of the collected data. Therefore, compensating for this interference is crucial to ensure reliable detection results [4,5,6,7].

The foundation for modeling aeromagnetic interference was established by Tolles and Lawson in 1950 [8]. Their model, commonly referred to as the T-L model, categorizes the interference magnetic field into three components: (1) the permanent magnetic field, (2) the induced magnetic field, and (3) the eddy current magnetic field. Over time, the T-L model has become the standard framework for aeromagnetic compensation [9]. In 1961, Leliak introduced a calibration flight scheme to estimate the model’s compensation coefficients, a method that remains in use today [10].

Despite its widespread adoption, the traditional T-L model faces several limitations. For instance, its 18 compensation coefficients are prone to multicollinearity, which can compromise the accuracy of the solution. To address this, Ma [11] proposed a method combining recursive least squares with elastic weights, effectively mitigating multicollinearity. Similarly, Zhang [12] employed principal component analysis to reduce multicollinearity. Yuan [13] highlighted the impact of aircraft non-rigidity on compensation accuracy and suggested incorporating additional fluxgate sensors to account for the minor displacements of moving parts. Ge [14] further improved the model by considering geomagnetic gradient effects, using the Earth’s magnetic moment and geographic coordinates to calculate the geomagnetic field at each point, thereby reducing errors caused by altitude variations.

While Leliak’s calibration scheme has seen limited updates over the years, it is increasingly inadequate for modern aeromagnetic platforms, particularly large unmanned aerial vehicles (UAVs). Liu [15] enhanced the traditional calibration flight scheme by simplifying maneuvers and reducing the flight area, thereby improving the robustness and effectiveness of the compensation. Yuan [16] recognized the influence of aircraft attitude on the compensation accuracy and proposed a straight-line flight scheme for small UAVs.

Given the growing use of large UAVs in aeromagnetic exploration, it is imperative to address the shortcomings of the T-L model, such as multicollinearity and geomagnetic gradient effects, and to develop new calibration flight schemes tailored to these platforms. Only through such advancements can the current demands of UAV-based magnetic exploration be met effectively.

In this study, we present a comprehensive modification of the traditional Tolles-Lawson (T-L) model to address two critical issues, including the coupling effect between the geomagnetic field and aircraft maneuvers, which cannot be fully eliminated by traditional filtering techniques, and the minor deformations that the aircraft fuselage undergoes during flight, due to its non-rigidity, that generate additional interfering magnetic fields. By expanding the original 18 compensation coefficients to 57 through a combination of term deletion and dynamic parameterization, the enhanced model achieves superior compensation accuracy.

Furthermore, recognizing the operational challenges associated with large unmanned aerial vehicles (UAVs) in traditional calibration flight schemes, we propose a novel compensation flight circle maneuver scheme. This scheme eliminates the yaw maneuver, which is prone to inducing operational errors and structural risks, and replaces it with an eight-sided flight path. Each side of the path incorporates roll and pitch maneuvers, significantly reducing the overall flight distance while maintaining compensation efficacy. The proposed scheme not only enhances the safety and stability of large UAVs during calibration flights but also minimizes the risk of structural damage and measurement inaccuracies caused by excessive maneuvering.

The feasibility and effectiveness of the proposed model and flight scheme are rigorously validated through experimental trials, demonstrating significant improvements in both compensation performance and operational safety.

## 2. Principles and Methods

### 2.1. T-L Model

**Figure 1:**
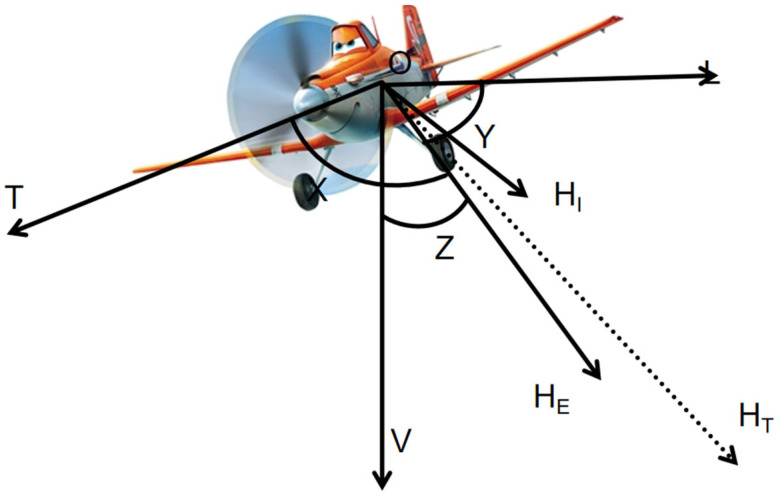
Aircraft reference coordinate system.

In 1950, Tolles and Lawson introduced a pioneering model, commonly referred to as the T-L model [8], which provides a concise framework for characterizing the magnetic interference generated by aircraft during aeromagnetic surveys. This model categorizes the interference magnetic field into three distinct components: the permanent magnetic field, the induced magnetic field, and the eddy current magnetic field. By leveraging this model, the interference magnetic field can be effectively reconstructed and compensated for during aeromagnetic exploration. Through the calculation of this model, the interfering magnetic field in the process of aviation magnetic exploration can be inverted very well. Firstly, we introduce the coordinate system in the T-L model, the coordinate system is established based on the aircraft flying in the air. As shown in Figure 1, the O point is the origin of the whole coordinate system, located in the center of the aircraft; the T axis is parallel to the fuselage, pointing to the nose; the L axis is parallel to the wing; the V is perpendicular to the belly of the fuselage, pointing to the ground; H_E_ represents the total geomagnetic field; and the direction is pointing to the center of the earth. The angle between the geomagnetic field H_E_ and the T-axis is defined as X; the angle between the geomagnetic field H_E_ and the L-axis is defined as Y; and the angle between the geomagnetic field H_E_ and the V-axis is defined as Z. H_T_ is the vector sum of the total magnetic field consisting of the T, L, and V-axes, and we can see that the total magnetic field H_T_ and the geomagnetic field H_E_ are inconsistent; the reason for this is that the total magnetic field H_T_ contains the geomagnetic field as well as the interference magnetic field H_I_. The formula is as follows:

(1) $$H_{T}=H_{E}+H_{I}$$

In the actual flight experiment, we measured the total magnetic field data as H_T_, of which the geomagnetic field part occupies most of the signal, while the interference magnetic field generated in the aeromagnetic exploration is a non-negligible part.

(2) $$cosX=\frac{H_{1}}{\sqrt{H_{1}^{2}+H_{2}^{2}+H_{3}^{2}}}$$

(3) $$cosY=\frac{H_{2}}{\sqrt{H_{1}^{2}+H_{2}^{2}+H_{3}^{2}}}$$

(4) $$cosZ=\frac{H_{3}}{\sqrt{H_{1}^{2}+H_{2}^{2}+H_{3}^{2}}}$$

$H_{1}$, $H_{2}$, $H_{3}$ represent the data obtained from the three axes T, L, V of the fluxgate vector magnetometer sensor, respectively.

(5) $$H_{T}=\sqrt{H_{1}^{2}+H_{2}^{2}+H_{3}^{2}}$$

In the T-L model, the interference magnetic field is divided into three parts: the permanent magnetic field, the induced magnetic field, and the eddy current magnetic field.

(6) $$H_{I}=H_{pe}+H_{in}+H_{ed}\;=p_{1}cosX+p_{2}cosY+p_{3}cosZ+H_{e}u_{11}{cos}^{2}X+H_{e}u_{22}{cos}^{2}Y+H_{e}u_{33}{cos}^{2}Z+H_{e}(u_{12}+u_{21})cosXcosY\;\;+H_{e}(u_{13}+u_{31})cosXcosZ+H_{e}(u_{23}+u_{32})cosYcosZ+H_{e}r_{11}cosXcos\dot{X}+H_{e}r_{12}cosYcos\dot{X}\;\;+H_{e}r_{13}cosZcos\dot{X}+H_{e}r_{11}cosXcos\dot{X}+H_{e}r_{21}cosXcos\dot{Y}+H_{e}r_{22}cosYcos\dot{Y}+H_{e}r_{23}cosZcos\dot{Y}\;\;+H_{e}r_{31}cosXcos\dot{Z}+H_{e}r_{32}cosYcos\dot{Z}+H_{e}r_{33}cosZcos\dot{Z}=\sum_{i=1}^{3}p_{i}c_{i}+H_{e}\sum_{i=1}^{3}\sum_{j=1}^{3}u_{ij}c_{i}c_{j}+H_{e}\sum_{i=1}^{3}\sum_{j=1}^{3}r_{ij}{\dot{c}}_{i}c_{j}$$

Here, we use $c_{i}$ to represent the cosine of the direction in the equation, i.e., the cosX,cosY, cosZ derivatives with respect to time. $p_{i}$ represent the coefficients of the permanent field. $u_{ij}$ represent the coefficients of the induced field. $r_{ij}$ represent the coefficients of the vortex field. Then, there are 18 compensation coefficients to be found at this point.

### 2.2. Modified T-L Model

Based on the relationship between the direction cosines, we can obtain the following equation:

(7) $${cos}^{2}X+{cos}^{2}Y+{cos}^{2}Z=1\;cosZcos\dot{Z}+cosYcos\dot{Y}+cosXcos\dot{X}=0$$

Consequently, the original 18 coefficients can be reduced to 16, significantly mitigating multicollinearity in the equations. This reduction enhances the accuracy of the solution and minimizes fitting errors in the interference magnetic field caused by computational inaccuracies.

In the T-L model, it is assumed that the aircraft’s fuselage structure and onboard equipment remain stable during flight maneuvers, without generating additional displacements due to changes in maneuvers. However, in actual flight, due to factors such as wind speed and turning maneuvers, even seemingly rigid objects can undergo minor deformations. As a result, the compensation coefficients $C_{i}$ will vary with changes in heading and need to be dynamically adjusted, which means the traditional use of the T-L model can not be a complete response to the interference magnetic field generated in the actual flight process [17]. Therefore, we need to make some corrections to the T-L so that it is better suited to the actual use of the environment.

Assuming that the compensation coefficient $C_{i}$ varies periodically with the heading angle θ and can be approximated by a first-order Fourier series, we obtain the following:

(8) $$C_{i}=\alpha_{i}+\beta_{i}cos\theta +\gamma_{i}sin\theta$$

$\alpha_{i}$ is the static component corresponding to the fixed coefficients in the original T-L model. $\beta_{i}$, $\gamma_{i}$ are the dynamic components that capture the influence of the heading angle.

The variation of the heading angle θ is periodic (0° to 360°), making the Fourier series a natural choice. A first-order approximation (containing only cosθ and sinθ) achieves a balance between computational complexity and model accuracy. Deformations such as wing bending can cause the magnetic moment distribution to change with heading, and the dynamic coefficients can capture this non-rigid effect.

Then, the original T-L equation should be written in the following form:

(9) $$H_{I}=H_{pe}+H_{in}+H_{ed}\;\;=\sum_{i=1}^{3}{(\alpha }_{i}+\beta_{i}cos\theta +\gamma_{i}sin\theta )c_{i}+H_{e}\sum_{i=1}^{3}\sum_{j=1}^{3}{(\alpha }_{i}+\beta_{i}cos\theta +\gamma_{i}sin\theta )c_{i}c_{j}\;\;+H_{e}\sum_{i=1}^{3}\sum_{j=1}^{3}{(\alpha }_{i}+\beta_{i}cos\theta +\gamma_{i}sin\theta ){\dot{c}}_{i}c_{j}$$

Here, we use $c_{i}$ to represent the cosine of the direction in the equation, i.e., the cosX,cosY, cosZ derivatives with respect to time, and $cos\theta =\frac{H_{2}}{\sqrt{H_{1}^{2}+H_{2}^{2}}},$ $sin\theta =\frac{H_{1}}{\sqrt{H_{1}^{2}+H_{2}^{2}}};$ $H_{1}$, $H_{2}$ represent the data obtained from the fluxgate vector magnetometer sensor triaxial T-L. The newly added $\beta_{i}cos\theta$ and $\gamma_{i}sin\theta$ terms explicitly model the coupling effect between the basis functions and the heading. When the aircraft turns, the direction cosine cosX,cosY, cosZ and the heading angle θ jointly affect the interference intensity, and the expanded terms can accurately capture this relationship.

At this stage, the number of compensation coefficients to be determined increases to 48. These coefficients are derived from the directional cosine vector, which is computed using triaxial data acquired from the three-axis fluxgate vector magnetometer. Each sample yields 48 elements that encapsulate the aircraft’s attitude information. Given that aeromagnetic surveys typically involve hundreds of thousands of samples, these elements collectively form a large matrix. Simultaneously, scalar magnetic field data are collected using an optical pump magnetometer. By solving this matrix, the 48 compensation coefficients can be estimated. Once these coefficients are determined, they are applied to the directional cosine matrix to fit the instantaneous interference magnetic field.

To evaluate the effectiveness of the compensation, Hardwick [18] introduced two key metrics: the standard deviation (STD) and the improvement ratio (IR). The STD serves as a quantitative measure of the residual magnetic interference after compensation, providing a means to assess the performance of the aeromagnetic compensation system. The IR, defined as the ratio of the STD before and after compensation, quantifies the improvement in compensation efficacy. A higher IR value indicates a more effective compensation process, reflecting a greater reduction in the magnetic interference.

(10) $$STD=\sqrt{\frac{1}{N}\sum_{i=1}^{n}{\left(X_{i}-\mu \right)}^{2}}$$

(11) $$IR=\frac{STD\left(H_{b}\right)}{STD\left(H_{a}\right)}$$

where $X_{i}$ are the data for each sampling point, μ is the mean value. STD is the squared difference of the data, which is a good reflection of the overall picture. $H_{b}$, $H_{a}$ represent the total magnetic field data collected by the optical pump before and after compensation, respectively. We have discussed above that the optical pump total field data contain both interference and geomagnetic signals. Then, when we use the optical pump total field data later, we need to consider using a band-pass filter to remove the contained geomagnetic field, so that we can obtain a pure interference field, with which it is easy to use the T-L model for aeromagnetic compensation.

(12) $$H_{T}=H_{e}+H_{I}=H_{e}+H_{pe}+H_{in}+H_{ed}=H_{e}+UC$$

where U is the direction cosine, and C is the compensation factor.

(13) $$bpf(H_{T})=bpf(H_{e})+bpf(U)C$$

After passing through the filter, if the geomagnetic field at this time is stable at a certain frequency, then $bpf(H_{e})$ = 0. However, due to the non-ideal nature of the calibration maneuver, the non-linearity of the filter and the non-uniformity of the geomagnetic field cause the aircraft to couple with the geomagnetic field during maneuvering to generate additional geomagnetic interference signals because the frequency bands are fused to the frequency of signals generated by maneuvering actions, so that the effects caused by the geomagnetic field cannot be completely filtered by the ordinary filter [13,19,20]. Dou proposed $bpf(H_{e})$ so that the residual geomagnetic field signal can be represented by the X, Y, Z axes [19].

(14) $$\begin{array}{rr} bpf\left(H_{E}\right) & =g_{x}cos\alpha \cdot bpf\left(T\right)+g_{y}cos\beta \cdot bpf\left(L\right)\\ & +g_{z}cos\gamma \cdot bpf\left(V\right) \end{array}$$

Written differently, by using Taylor series expansion, we can consider the filtered residual magnetic field as components in both horizontal and vertical directions and, ultimately, use longitude and latitude to construct a horizontal-component ground field model and height to construct a vertical-component geomagnetic field model, so that the filtered residual magnetic field $\mathrm{bpf}(H_{E})$ can be roughly fit by the fitting of the three coefficients.

(15) $$bpf(H_{e})=bpf(W)a+bpf(J)b+bpf(L)c$$

The T-l model can then be changed to the following:

(16) $$H_{IE}=H_{pe}+H_{in}+H_{ed}+H_{e}\;=\Sigma_{i=1}^{3}{(\alpha }_{i}+\beta_{i}cos\theta +\gamma_{i}sin\theta )c_{i}+H_{e}\Sigma_{i=1}^{3}\Sigma_{j=1}^{3}{(\alpha }_{i}+\beta_{i}cos\theta +\gamma_{i}sin\theta )c_{i}c_{j}+H_{e}\Sigma_{i=1}^{3}\Sigma_{j=1}^{3}{(\alpha }_{i}+\beta_{i}cos\theta +\;\;\gamma_{i}sin\theta ){\dot{c}}_{i}c_{j}+{(\alpha }_{1}+\beta_{1}cos\theta +\gamma_{1}sin\theta )W+{(\alpha }_{2}+\beta_{2}cos\theta +\gamma_{2}sin\theta )J+{(\alpha }_{3}+\beta_{3}cos\theta +\gamma_{3}sin\theta )L$$

where $c_{i}$ represent the cosine of the direction in the equation, i.e., the cosX,cosY, cosZ derivatives with respect to time. W represents the latitude vector, and J represents the longitude vector, thus totaling from 48 compensation coefficients to 57 compensation coefficients to be solved.

Through such a model modification to minimize the residual geomagnetic field interference formed by the coupling of maneuvering actions, the interference magnetic field during the aeromagnetic detection process can be better fitted to the real situation, and the actual interference magnetic field obtained by the optical pump is subtracted from the interference magnetic field fitted by the improved T-L model, and what is obtained in this way is the compensated aeromagnetic detection signal. The effect of compensation determines the ease of signal target recognition, so the excellent compensation mechanism is very meaningful; while the T-L model can only solve part of the compensation optimization effect, the details of the compensation still need to modify the model.

### 2.3. Modified T-L Model Effect

To validate the efficacy of the improved model, a series of experimental flights were conducted in the East China Sea, Zhejiang Province, China. A small unmanned aerial vehicle (UAV) equipped with an optical pump magnetometer, a triaxial fluxgate magnetometer, a GPS module, and an inertial navigation system was deployed for magnetic field detection. The calibration flights followed the traditional scheme, involving sequential yaw, pitch, and roll maneuvers on all four sides of a predefined flight path. Additionally, level flight data from the surrounding area were collected to test the compensation performance.

**Figure 2:**
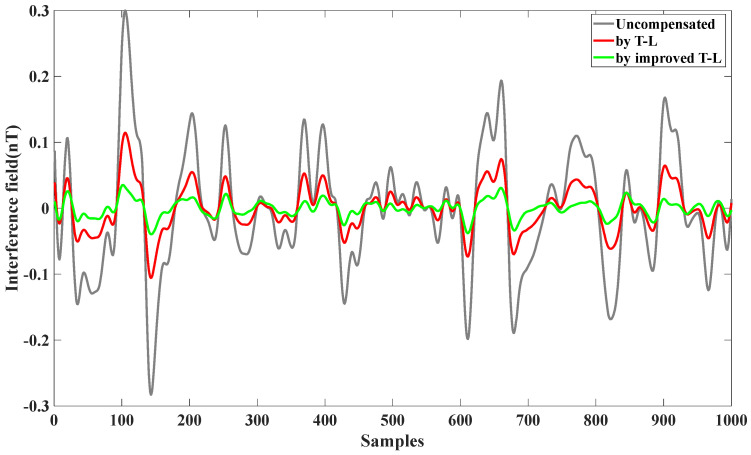
Compensation of level flight data using the T-L model and improved T-L model.

During the calibration flights, scalar magnetic field data were acquired using the optical pump magnetometer, while triaxial vector data were recorded by the fluxgate magnetometer. Both the traditional T-L model and the improved T-L model were applied to compensate for the magnetic interference. The results, illustrated in Figure 2, demonstrate the comparative effectiveness of the two models in reducing residual magnetic interference.

From Table 1, we can see the effect of the compensation; the same data before compensation for the interference magnetic field of 0.1131 nT, using the T-L model compensation, were reduced to 0.0184 nT; with the improved T-L model, they were reduced to 0.0164 nT. The improvement ratio IR increased by 24.41%; it can be seen that the effect is perfect.

### 2.4. Improved Compensation Flight Circle

Following the introduction of the Tolles-Lawson (T-L) model, which provided a mathematical framework for fitting magnetic interference noise, Leliak proposed a calibration flight scheme in 1961 to compensate for magnetic field interference. This scheme, which remains widely used to this day, has proven highly effective in enhancing aeromagnetic compensation accuracy.

**Figure 3:**
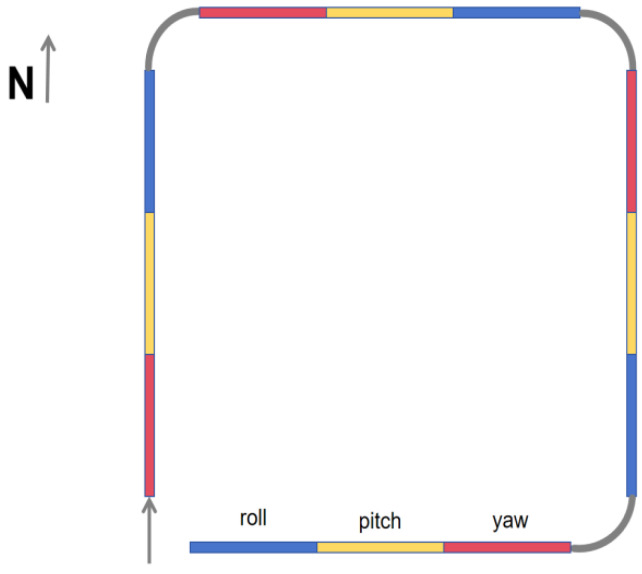
Flight paths traditionally used for compensation.

As illustrated in Figure 3, the traditional calibration flight protocol begins with the aircraft at a level flight attitude. The flight path follows a rectangular trajectory, proceeding sequentially from south to north, west to east, north to south, and finally east to west. On each leg of the path, the aircraft performs three specific maneuvers: yaw (±5°), pitch (±5°), and roll (±10°). These maneuvers must be executed consistently to ensure accurate compensation. Additionally, the distance of level flight between maneuvers is minimized to reduce the overall flight duration and optimize the compensation process.

The traditional calibration flight scheme presents several significant challenges:

1. **Operational Complexity and Equipment Displacement:**

During the flight, each leg of the path requires the execution of three consecutive maneuvers: yaw, pitch, and roll. These maneuvers are particularly challenging to perform accurately during high-speed flight. The continuous and rapid changes in aircraft attitude increase the likelihood of minor displacements between the aircraft and onboard equipment, such as triaxial fluxgate magnetometers. Such displacements generate additional interference magnetic fields that cannot be adequately modeled by the T-L model, leading to errors in the compensation results.

2. **Extended Flight Path and Geomagnetic Instability:**

The execution of yaw, pitch, and roll maneuvers necessitates a longer flight path to allow for gradual adjustments. This results in an expanded calibration flight area, which can encompass regions with unstable geomagnetic fields. Since the geomagnetic field significantly influences the total magnetic field data collected by the optical pump magnetometer, such instability can degrade the accuracy of the compensation process.

3. **Safety Risks for Large UAVs:**

**Figure 4:**
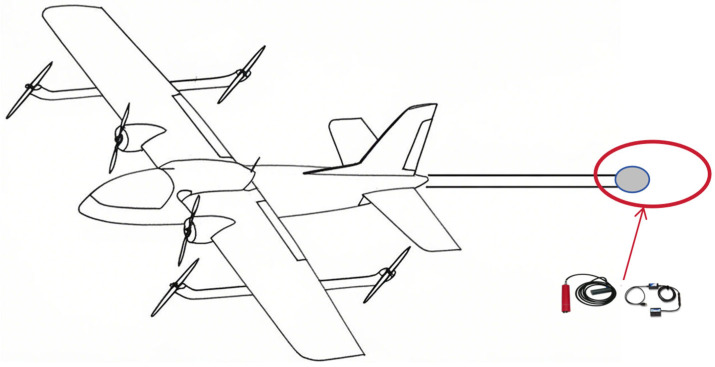
Schematic diagram of a large drone with an additional tail section for placing magnetic prospecting equipment; inside the red circle, the required magnetic prospecting instruments are placed, such as triaxial magnetometers and optically pumped magnetometers, in order to be far away from the magnetic noise interference of the fuselage.

Historically, aeromagnetic surveys were conducted using slower, manned aircraft, where the risks associated with high-speed maneuvering were minimal. With the advent of small UAVs, the risks remained manageable due to their compact size and agility. However, the current trend toward large UAVs, which are heavier and carry more payload, introduces significant safety concerns during high-speed maneuvers. Specifically, yaw maneuvers along the *X*-axis can induce substantial stress on the fuselage, increasing the risk of structural damage. In contrast, roll and pitch maneuvers, which primarily affect the *Y*- and *Z*-axes, pose less risk. Additionally, to minimize their impact on magnetic field measurements, optical pump magnetometers and triaxial fluxgate sensors are typically mounted far from the fuselage, often in an extended tail section (as shown in Figure 4). This configuration exacerbates the risk of instrument displacement or even structural failure during yaw maneuvers, generating additional interference magnetic fields that cannot be modeled by the T-L model. Consequently, these factors contribute to errors in the compensation results.

Given these challenges, it is imperative to optimize the calibration flight maneuvers for large UAVs by eliminating high-risk maneuvers, such as yaw, that have a detrimental impact on both safety and compensation accuracy.

First of all, it is essential to clarify the role of compensation maneuvers in aeromagnetic surveys. Taking yaw as an example, during flight, external factors such as the wind can induce yaw motions. If the compensation model fails to account for yaw, the compensation efficacy will deteriorate, and the resulting magnetic data will be adversely affected. By incorporating yaw maneuvers into the calibration process, the corresponding compensation data can be collected and integrated into the model. This enables the compensation system to effectively mitigate yaw-induced errors during actual flight operations.

**Figure 5:**
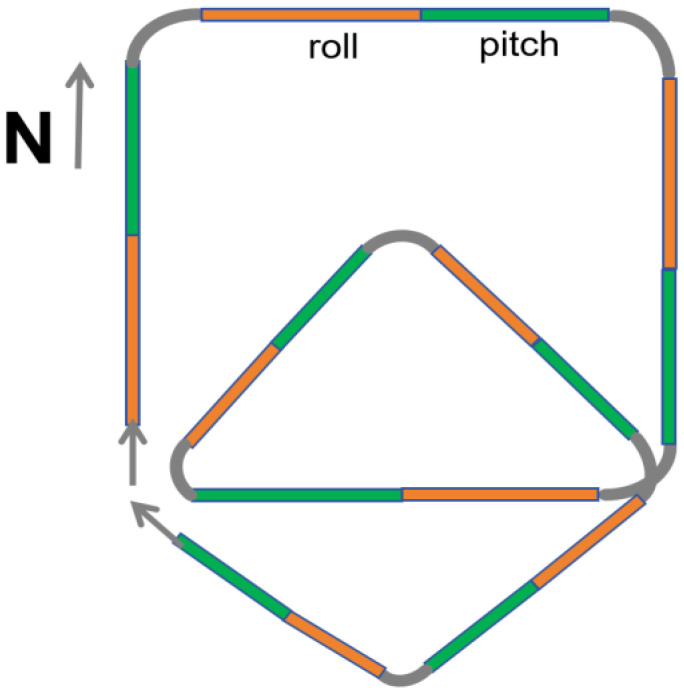
Improved flight path for aeromagnetic compensation.

However, based on the aforementioned analysis, we propose eliminating yaw maneuvers from the calibration process and retaining only pitch and roll maneuvers on each flight leg. While yaw is undeniably significant for compensation, its removal is justified by the associated risks and complexities. To address this, we introduce a novel calibration flight path, as illustrated in Figure 5.

The improved scheme involves the following steps:

1. The aircraft begins at a level flight attitude and sequentially performs pitch (±5°) and roll (±10°) maneuvers on each leg of the flight path.
2. The flight path consists of two overlapping quadrilaterals, forming an octagonal trajectory. The first quadrilateral is flown clockwise, followed by the second in a rhombic configuration.

This design offers several advantages over the traditional calibration flight scheme:

1. **Compensation for Yaw Effects:** Although yaw maneuvers are removed, the eight-sided flight path incorporates seven turns, effectively simulating yaw-induced magnetic interference and compensating for its absence in the calibration process.
2. **Enhanced Safety for Large UAVs:** By eliminating high-risk yaw maneuvers, the proposed scheme reduces the likelihood of structural damage or displacement of tail-mounted equipment, such as optical pump magnetometers and triaxial fluxgate sensors.
3. **Optimized Flight Efficiency:** The removal of yaw maneuvers shortens the flight distance for each leg. Additionally, the overlapping quadrilateral design minimizes the overall flight area, reducing the impact of geomagnetic gradient variations on the compensation process.

## 3. Experiments and Results

### 3.1. Compensation Flight Experiments

**Figure 6:**
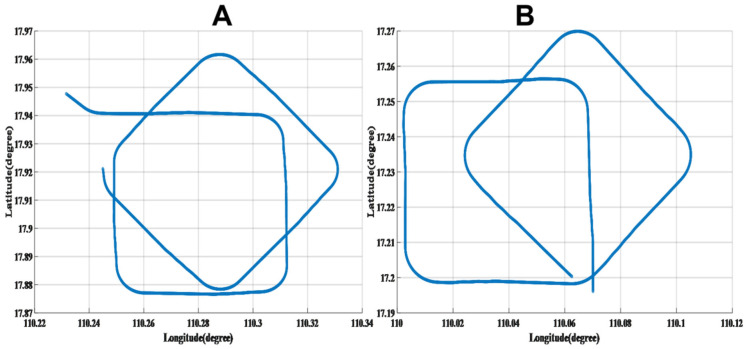
Calibration flight circle (**A**) and verification flight circle (**B**).

Experimental trials were conducted in the waters near Sanya, in the South China Sea, where two calibration missions, designated as A and B, were performed in adjacent areas, as depicted in Figure 6. The experiments utilized a state-of-the-art, large-scale magnetic prospecting unmanned aerial vehicle (UAV) equipped with an optical pump magnetometer, a triaxial fluxgate magnetometer, an inertial navigation system, a GPS module, and additional payload instruments. During flight, the optical pump magnetometer recorded scalar total magnetic field data, while the triaxial fluxgate magnetometer provided vector magnetic field data (*X*, *Y*, *Z*) in the aircraft coordinate system. The inertial navigation system captured Euler angles (pitch, roll, and yaw) during maneuvers, and the GPS module supplied real-time altitude, speed, longitude, and latitude data.

Mission A followed a flight path entering from the west to the east, subsequently departing the compensation area toward the northwest. Mission B entered from the south to the north and exited toward the southeast. The UAV operated at an altitude of approximately 3500 m and a speed of 50 m/s (180 km/h), with wind speeds reaching 10 m/s. These conditions posed significant challenges and risks for maneuvering a large UAV, underscoring the importance of the proposed flight scheme.

Data acquisition was conducted at a sampling frequency of 10 Hz. In accordance with the improved Tolles-Lawson (T-L) model, all scalar and vector data were preprocessed using a Butterworth filter with a bandwidth of 0.04–0.6 Hz to isolate relevant magnetic interference signals.

**Figure 7:**
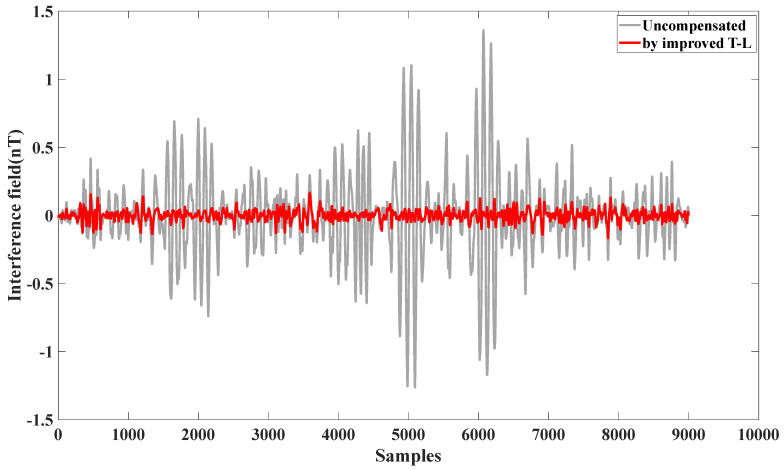
Effect of self-compensation using calibration flight circle A.

From Figure 7, it can be seen that the interference magnetic field generated by the obtained calibration flight circle A is compensated for by using the modified T-L model.

**Figure 8:**
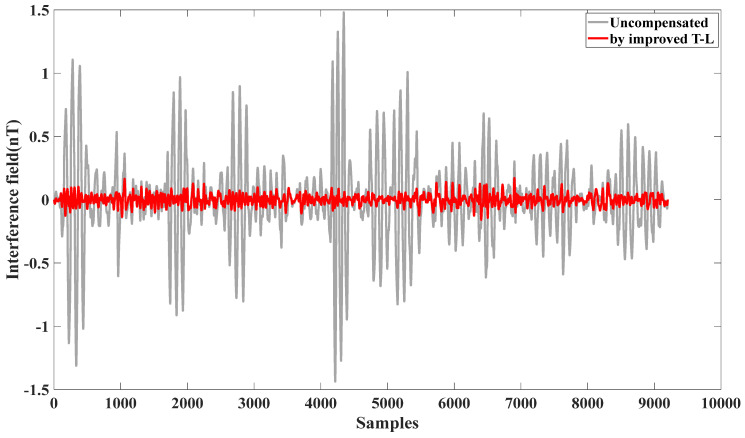
Effect of self-compensation using verification flight circle B.

From Figure 8, it can be seen that, by using the modified T-L model, the obtained verification flight circle B generates an interfering magnetic field that compensates for itself.

The compensation results demonstrate the effectiveness of the self-compensation process. As summarized in Table 2, the interference magnetic field generated during calibration flight circle A was measured at 0.2912 nT (standard deviation, STD). After applying the improved Tolles-Lawson (T-L) model, the interference was reduced to 0.0404 nT, achieving an improvement ratio (IR) of 7.3171. These results validate that the proposed calibration flight scheme meets the aeromagnetic compensation requirements for this UAV platform. Similarly, for calibration flight circle B, the interference magnetic field was reduced from 0.2912 nT to 0.0404 nT, with an IR of 7.834.

**Figure 9:**
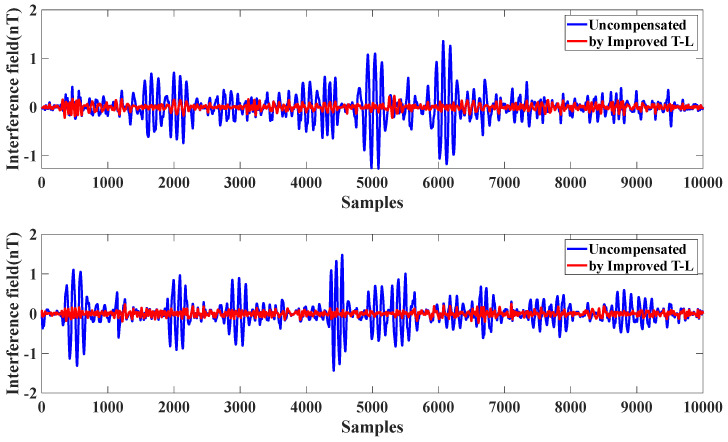
The compensation effect of mutual cross-interference between A and B.

To further evaluate the robustness of the model, the compensation effects were assessed using cross-validation between calibration flight circle A and verification flight circle B. As shown in Figure 9, the upper subplot illustrates the compensation effect of circle A on circle B, while the lower subplot shows the compensation effect of circle B on circle A. As summarized in Table 2, the interference magnetic field in circle A was reduced from 0.2912 nT to 0.0436 nT, achieving an improvement ratio (IR) of 6.6789. Similarly, the interference magnetic field in circle B was reduced from 0.3264 nT to 0.0465 nT, also achieving an IR of 7.0194. This cross-validation confirms the reliability and generalizability of the proposed compensation framework.

The cross-compensation effect of flight circles A and B is shown in Table 3, both of which have a significant compensation effect on noise.

**Figure 10:**
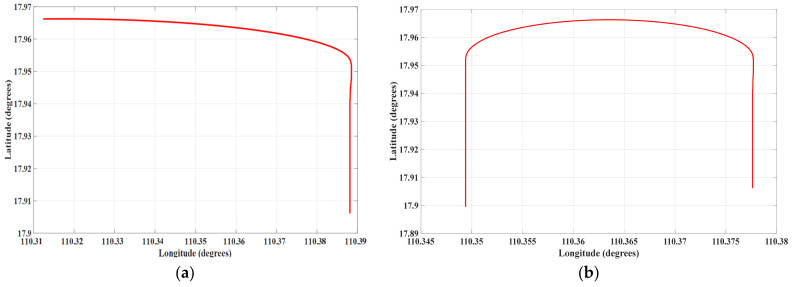
Level flight path C and level flight path D during flight: (**a**) flight **C**, (**b**) flight D.

During the flight, a segment of level flight was analyzed, with two distinct sections, denoted as path C and path D, extracted for evaluation (Figure 10). Path C features a sharp turn, while path D includes a gentle turn.

**Figure 11:**
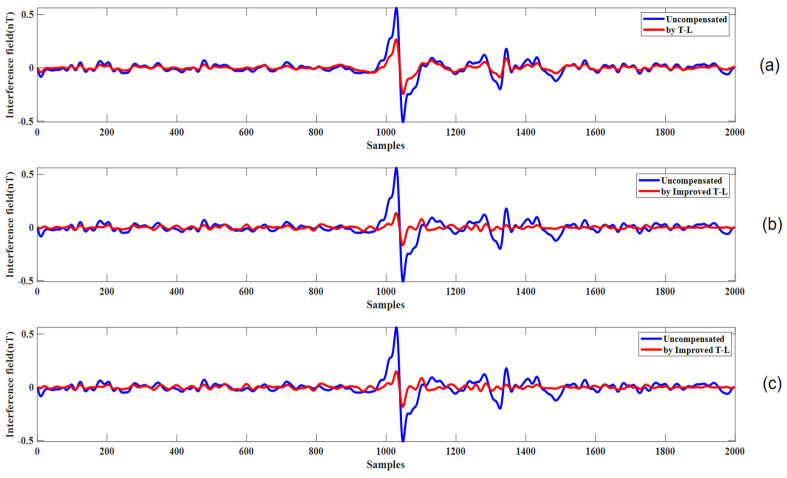
(**a**) The compensation results for level flight path C using the traditional Tolles-Lawson (T-L) model. (**b**) The compensation results for level flight path C using the improved T-L model with coefficients derived from calibration flight circle A. (**c**) The compensation results for level flight path C using the improved T-L model with coefficients derived from verification flight circle B.

The compensation results for level flight path C, using coefficients derived from calibration circle A and verification circle B, are presented in Figure 11b and c, respectively. The sharp turn in path C generates a significant anomalous signal due to abrupt aircraft maneuvers. However, the improved Tolles-Lawson (T-L) model effectively eliminates this anomaly, while also providing partial compensation for the remaining level flight segments.

Figure 11a presents the compensation effect for the level flight path C using the compensation coefficients derived from the calibration flight circle A and the traditional Tolles-Lawson (T-L) model. The figure shows that the anomalous signal generated by the turn is not well handled.

**Figure 12:**
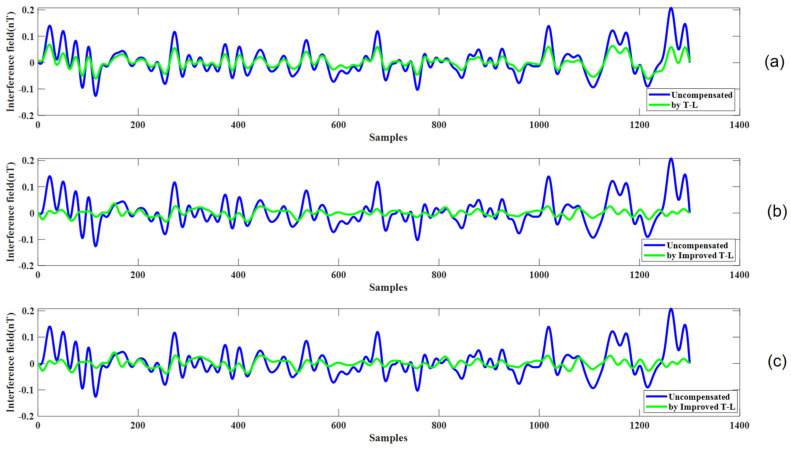
(**a**) The compensation results for level flight path D using the traditional Tolles-Lawson (T-L) model. (**b**) The compensation results for level flight path D using the improved T-L model with coefficients derived from calibration flight circle A. (**c**) The compensation results for level flight path D using the improved T-L model with coefficients derived from verification flight circle B.

Similarly, Figure 12b,c illustrate the compensation results for level flight phase D, using coefficients from calibration circle A and verification circle B, respectively. In contrast to path C, the turn in path D is smoother, resulting in less pronounced anomalous signals. Although the maneuvering effects are less severe, the extended duration of the turn phase highlights the model’s ability to handle prolonged disturbances.

Figure 12a shows the compensation effect for the level flight path D using the compensation coefficients derived from the calibration flight circle A and the traditional Tolles-Lawson (T-L) model. The figure illustrates that the compensation effect is not as good as that of the improved model.

The flight data A, B were used to solve for the compensation coefficients, to be brought in later, for C, D in the process of level flight. As shown in Table 4, when the flight data of A were used to solve the compensation coefficients for C to carry out aeromagnetic compensation, the interfering magnetic field was reduced from 0.1605 nT down to 0.0385 nT, with the improvement ratio IR = 4.1688, and the aeromagnetic compensation for D reduced the interference field from 0.0529 nT to 0.0246 nT, with an improvement ratio of IR = 2.1504. When the compensation coefficients were solved from the flight data of B, the aeromagnetic compensation of C reduced the interfering magnetic field from 0.1605 nT to 0.0398 nT, with an improvement ratio of IR = 4.0428, and the aeromagnetic compensation of D reduced the interfering magnetic field from 0.0529 nT to 0.0256 nT, with an improvement ratio of IR = 2.0664.

The improvement ratio (IR) was significantly enhanced through the model’s improvement. As can be seen from the comparison between Table 4 and Table 5, the improvement ratio (IR) for level flight phase C increased by 80% when using the improved model, and the improvement ratio for level flight phase D increased by 22.76%.

### 3.2. Result Analysis

To validate the rationale behind our proposed improvements to the compensation flight maneuvers, we conducted two calibration flights, designated as A and B, to acquire the necessary data. As shown in Table 2, the compensation coefficients derived from these flights effectively reduced the interference magnetic field when applied to the same datasets, demonstrating the model’s self-consistency. Furthermore, Figure 9 and Table 3 illustrate that the coefficients obtained from flight A successfully compensated for the interference in flight B, achieving a significant improvement ratio (IR) of 6.6789, and the coefficients obtained from flight B successfully compensated for the interference in flight A, achieving a significant improvement ratio (IR) of 7.0194. This cross-validation confirms the robustness of the proposed method.

Given that level flight constitutes the majority of actual flight operations, we applied the compensation coefficients from flights A and B to level flight segments C and D. The results, summarized in Table 4, indicate satisfactory compensation performance. However, as depicted in Figure 11 and Figure 12, the compensation effect during level flight was relatively modest. This is attributed to the limited magnetic interference generated during steady, non-maneuvering flight, which poses a challenge for the T-L model to achieve pronounced improvements. Nevertheless, the model demonstrated exceptional efficacy in compensating for interference during the turning phases, where maneuvering-induced magnetic disturbances are more significant. Meanwhile, Table 5 also shows that the improved compensation model made significant progress compared with the traditional model.

In conclusion, the modified calibration flight scheme is well suited for large UAVs, offering compensation performance comparable with that of traditional methods [21,22,23] while addressing their inherent limitations. The proposed improvements not only enhance the safety and feasibility of calibration flights for large UAVs but also provide a robust framework for mitigating interference noise in real-world operational scenarios.

## 4. Conclusions

In aeromagnetic surveys, enhancing the target detection accuracy necessitates the reduction of magnetic interference generated by the aircraft itself. Consequently, magnetic interference compensation is a critical step in data processing. Traditional compensation methods often fail to account for two critical issues, including the coupling effect between the geomagnetic field and aircraft maneuvers, which cannot be fully eliminated by traditional filtering techniques, and the problem of non-rigidity of the aircraft fuselage during flight, which causes minor deformations and generates additional interfering fields.

To address these limitations, this study introduced an enhanced model based on the traditional Tolles-Lawson (T-L) framework. The proposed model expanded the original 18 compensation coefficients to 57, incorporating dynamic adjustments for improved accuracy. Experimental validation demonstrated a 24.41% increase in the improvement ratio (IR) compared with the conventional T-L model, highlighting its superior performance.

Furthermore, recognizing the operational risks associated with large unmanned aerial vehicles (UAVs), we proposed a modified calibration flight scheme. By eliminating the yaw maneuver and optimizing the flight path, the revised scheme significantly reduced the risks and complexities of aeromagnetic compensation for large UAVs. Validation tests confirmed the effectiveness of this approach, when compensating for interference noise during level flight, the best IR achieved was 4.1688. Meanwhile, by comparing the traditional T-L model with the improved model under the same data and compensation flight scheme, the IR of the improved model increased by up to 80%. These results underscore the model’s exceptional compensation performance for large UAVs.

In summary, the proposed improvements provide a safer, more efficient, and highly effective framework for aeromagnetic compensation, particularly suited to the operational demands of large UAVs.

## Figures and Tables

**Table 1:** Compensation comparison using the T-L model and the modified T-L model.

Method	Uncompensated (std)	Compensated (std)	IR
T-L	0.1131 (nT)	0.0229 (nT)	4.9408
Improved T-L	0.1131 (nT)	0.0184 (nT)	6.1467

**Table 2:** Effect of self-compensation using calibration flight circle A and verification flight circle B.

Flight	Uncompensated (std)	Compensated (std)	IR
A	0.2912 (nT)	0.0404 (nT)	7.3173
B	0.3264 (nT)	0.0415 (nT)	7.8634

**Table 3:** The compensation effect of cross-interference between flight circle A and flight circle B.

Compensation Target	Uncompensated (std)	Compensated (std)	IR
A → B	0.2912 (nT)	0.0436 (nT)	6.6789
B → A	0.3264 (nT)	0.0465 (nT)	7.0194

**Table 4:** The effect of using flight data A, B to obtain compensation coefficients to compensate for C, D level flight.

Flight	Compensated Object	Uncompensated (std)	Compensated (std)	IR
A	Flight C	0.1605 (nT)	0.0385 (nT)	4.1688
	Flight D	0.0529 (nT)	0.0246 (nT)	2.1504
B	Flight C	0.1605 (nT)	0.0398 (nT)	4.0428
	Flight D	0.0529 (nT)	0.0256 (nT)	2.0664

**Table 5:** Using calibration flight A, the traditional T-L model compensates for noise interference in level flights C and D.

Compensation Target	Uncompensated (std)	Compensated (std)	IR
A → C	0.1605 (nT)	0.0696 (nT)	2.3060
A → D	0.0529 (nT)	0.0302 (nT)	1.7517

## Data Availability

The data presented in this study are available on request from the corresponding author because the data contain confidential information by design in a very special way.
